# Drug Policy Research & Pharmacoeconomics

**Published:** 2008-10

**491**

**Anti-microbial drug utilization in patients of febrile neutropenia on cancer chemotherapy**

Saxena D, Roy V, Bahadur A K, Mishra B

**Maulana Azad Medical College and Loknayak Hospital, New Delhi, India.**

**Background:**

Chemotherapy induced febrile neutropenia(FN), is a serious side effect of cancer treatment requiring the judicious use of antimicrobial agents(AMA). Irrational use of broad spectrum antibiotics may result in emergence of resistant strains, superinfections, increased ADR’s and wasteful expenditure. The objective of the study was to assess the pattern of use of AMA in FN patients.

**Materials and Methods:**

An Observational, Prospective Cohort study was conducted. Patients suffering from cancer with febrile neutropenia were assessed for the treatment administered specifically for the use of AMA, granulocyte colony stimulating factor (G-CSF) and paracetamol (PCM). Patients were categorised in low/high risk categories based on IDSA guidelines. The time to defervesence and recovery of absolute neutrophil count (ANC) was observed.

**Results:**

A total of 211 patients were enrolled in the study. The incidence of FN in our hospital was 13.3%. Majority were low risk (98.1%) patients. The average antimicrobial exposure in days per patient was 4.16 days and per FNE 3.5 days. The AM exposure density was 1.26. A total of 8 antimicrobial agents were used, five anti-bacterial, one anti-anaerobic, one anti-fungal and antiviral drug. These were Ciprofloxacin (52.1%), Ceftriaxone(37.9%), Cefuroxime(3.7%), Cefoperazone-Sulbactam(5.2%), Amoxicillin- Clavulanic acid (0.9%). Metronidazole was administered to all patients. All patients received combination AM and G-CSF irrespective of risk status. No correlation between PCM use and time to defervesence was observed. The time to defervesence and time to recovery of ANC>100 cell/millilitre cube was maximum with Cefuroxime-Metronidazole combination.

**Conclusion:**

The selection of AM agents does not appear to be based on specific patient factors, risk category or antibiotic sensitivity pattern. There is excessive use of G-CSF. Guidelines for appropriate use of antimicrobials need to be formulated for treatment of FN due to cancer chemotherapy in our hospital.

**492**

**Pharmacoeconomic study of psychotropic drug use in psychiatry outpatient department in a tertiary care teaching hospital in western India**

Goyal S, Samant B D, Parkar S R

**Seth GS Medical College and KEM Hospital, Mumbai, India.**

**Objective:**

Pharmacoeconomic evaluation of psychotropic drug use in Psychiatry OPD in a tertiary care teaching hospital in western India.

**Materials and Methods:**

The study was conducted after ethics committee approval. 300 patients of either sex and irrespective of age were included. Written informed consent was obtained. The data from the OPD card of the patient was copied on a structured performa. Price (calculated for 30 day) for drugs available on hospital schedule was calculated as per the price list available in hospital pharmacy. The cost of non scheduled drugs was calculated as per the Municipal corporation approved subsidized medical store price list. Socioeconomic score of the patients was calculated on the basis of Modified Kuppuswami Scale.

**Results:**

Average number of drugs per prescription was 2.53 ± 0.97 (758/300) and drugs from hospital pharmacy were 67.41%. Average cost of prescription was 117.39 INR. Average cost borne by hospital pharmacy was 17.01 INR and borne by patient was 100.38 INR. A spearman correlation demonstrated that total cost of the treatment was significantly directly correlated with the socioeconomic score of the patients [Spearman.s Rho =0.400, significance (2 tailed) =0.000] and cost borne by hospital pharmacy was significantly inversely correlated with the socioeconomic score of the patients [Spearman’s Rho = -0.279, significance (2 tailed) =0.000].

**Conclusion:**

Patients of lower socioeconomic score were given drugs mainly from hospital pharmacy. The average cost borne by patient is 6 times more as compared to cost of drugs dispensed by hospital pharmacy. This suggests that if non scheduled drugs are available on schedule, it can save the expenditure borne by the patient.

**493**

**Analysis of antibiotic prophylaxis within various surgical departments in tertiary hospital**

Anil Kumar MH, Niveditha, Natasha JP, Priyalatha, Shivamurthy MC

**M.S.Ramaiah Medical Collage, Bangalore, India.**

**Introduction:**

Surgical site infections (SSI) are the most commonly reported nosocomial infections. The incidence varies between 7-12%. They have been responsible for increased health care cost, morbidity and mortality related to surgical procedures. This can be minimized by administration of pre-operative antibiotics that produces higher concentration of the antibiotic in relevant tissues at the site of operation.

**Aim:**

The objective of the study was to analyze pre-operative antibiotics given for the surgical procedure was appropriate and in accordance with Hospital Infection Control Committee (HICC) antibiotic policy.

**Methods:**

The details of the patients who underwent surgical procedure from 1^st^ January to 31^st^ January 2008 were obtained retrospectively. The information included type of surgical procedure; pre operative antibiotic used, its dose, route of administration, time and duration of use. These details were compared with HICC guidelines.

**Results:**

482 operations were analyzed. The patient’s age ranged from 1 month to 87 years, 57% patients were male. Common antibiotics used were ceftriaxone, gentamicin and metronidazole. The pre-operative antibiotic guidelines were followed upto 90% in orthopedics, 89% in OBG, 87% in neuro surgery, 75% in general surgery.

**Conclusion:**

In this study, high level of concordance and appropriateness was observed in Orthopedics OBG and Neuro surgery departments. The deviations observed from the guidelines were in terms of time of administration and duration of the particular antibiotic used.

**494**

**Study of rationality of paracetamol combination with ibuprofen *and* nimesulide as antipyretic**

Anand M, Shukla AK, Chugh Y, Sharma A, Kumar S, Chaurasia RC, Kastury N

**Mln Medical College, Allahabad, India.**

**Background:**

*Paracetamol* alone is used as first choice antipyretic, but several combinations of *Paracetamol* with other non-steroidal anti-inflammatory drugs are available in the Indian market. Though the combinations have additional advantage of anti-inflammatory activity but they are used widely as antipyretic only.

**Objective:**

To evaluate the rationality of combinations of *paracetamol* with *ibuprofen / nimesulide* available in the Indian pharmaceutical market.

**Materials and Methods:**

list of marketed fixed dose combinations of *paracetamol* with *ibuprofen and with nimesulide* were assessed from drug indices namely drug today, idr, mims, cims, adr and drug updates. The combinations were grouped on the basis of their composition. The justification provided by the manufacturers, was critically reviewed and compared with evidence- based information from standard text namely goodman and gilman’s-the pharmacological basis of therapeutics and who essential medicine list; in order to label them as rational or irrational.

**Results:**

The pharmacokinetic profile of *paracetamol* does not match entirely with that of *ibuprofen* and *nimesulide*. Further there is lot of discrepancy in the doses of drugs combined and no such combinations are recommended in who essential medicine list.

**Conclusion:**

*Paracetamol* used alone is safest antipyretic and its combination with *ibuprofen and nimesulide* has no further added advantage in enhancing its antipyretic effect. Instead such fdcs not only increase the drug burden on patients but also increase the toxicity profile and cost of the product. Moreover, strict dose standardization must be followed to avoid the emerging confusion regarding the dose of drugs combined.

**495**

**A questionnaire based survey on knowledge, attitude and practice of medical practitioners about medicinal product promotional activities**

Datta A, Bhowmick S, Chatterjee S

**Institute of Postgraduate Medical Education and Research, Kolkata, India.**

**Introduction:**

WHO defines drug promotion as all informational and persuasive activities by manufacturers and distributors to induce/ influence the sale and use of medicines. It is conceived that drug promotional activities might have an important bearing on prescribing trends by medical practitioners. This questionnaire based survey was undertaken to evaluate the knowledge, attitude and practice (KAP) of doctors pertaining to drug promotion by the Indian pharmaceutical industry.

**Methods:**

A questionnaire was designed comprising both closed and open ended questions equally weighted to detect the KAP quotients and distributed to clinicians of teaching institutes. Anonymity of the respondent was maintained. Data was analyzed by descriptive statistical tools.

**Results:**

Of 250 forms distributed only 110 responded. Analysis revealed that although the knowledge and attitude of doctors to promotion varied but their practice or behavior were similar. Most respondents (92%) opined that promotion has an impact on their behavior. The main source of new drug information was from representatives of the industry which is biased and not always scientifically validated. Respondents were not aware of essential components in promotional material. Although majority (99%) accept inducements (pens, educational materials, conference sponsorship etc.) in various forms but only 34% stated that it influenced their prescribing habits.

**Conclusion:**

Most clinicians want strict implementation of ethical promotional norms in India which will improve the scientific content of promotional information, minimize aggressive promotion by the industry and facilitate cost reduction of medicines. The medical community should be sensitized to critically review promotional material and validate it before prescribing.

